# CPR and ECMO: The Next Frontier

**DOI:** 10.5041/RMMJ.10399

**Published:** 2020-04-29

**Authors:** Daniel I. Ambinder, Matt T. Oberdier, Daniel J. Miklin, Henry R. Halperin

**Affiliations:** 1 Department of Medicine, Johns Hopkins University School of Medicine; Baltimore, MD, USA; 2 Department of Medicine, University of Southern California, Los Angeles, CA, USA; 3 Department of Radiology, Johns Hopkins University School of Medicine; Baltimore, MD, USA; 4 Department of Biomedical Engineering, Johns Hopkins University School of Medicine; Baltimore, MD, USA

**Keywords:** CPR, ECMO, hemodynamics, resuscitation, sudden cardiac arrest

## Abstract

Cardiopulmonary resuscitation (CPR) is a first-line therapy for sudden cardiac arrest, while extracorporeal membrane oxygenation (ECMO) has traditionally been used as a means of countering circulatory failure. However, new advances dictate that CPR and ECMO could be complementary for support after cardiac arrest. This review details the emerging science, technology, and clinical application that are enabling the new paradigm of these iconic circulatory support modalities in the setting of cardiac arrest.

## INTRODUCTION

### Sudden Cardiac Arrest

Cardiac arrest is defined as the loss of mechanical activity of the heart, leading to the sudden loss of forward blood flow. This causes a marked reduction or elimination of perfusion to vital organs, which can rapidly lead to death if circulation is not restored. There are over 350,000 out-of-hospital and over 205,000 in-hospital cardiac arrest events each year in the United States,^1^ with sudden cardiac arrest accounting for more than 60% of all cardiac deaths.^2^ The initial rhythms that present with cardiac arrest include ventricular fibrillation, pulseless ventricular tachycardia, pulseless electrical activity (normal electrical activity but no or minimal cardiac function), and asystole.^1^ The initial approach to all of these rhythms includes initiation of cardiopulmonary circulation.

### CPR—Strengths and Limitations

A major guiding principle of resuscitation is that increasing the amount of forward blood flow generated during resuscitation increases chance of survival.^3^–^5^ The combination of cardiopulmonary resuscitation (CPR) and defibrillation for shockable rhythms (ventricular fibrillation and pulseless ventricular tachycardia) can eliminate lethal arrhythmias and restore blood flow during resuscitation. A major component of CPR is application of chest compressions to increase intrathoracic pressure and, in turn, increase mean blood pressure and forward blood flow. However, the quality of compressions provided can vary based on a provider’s physical capabilities. Rescuer fatigue is a limitation of manual CPR,^6^,^7^ but can be mitigated by frequently changing providers, or, when available, using an automated device, which may also provide more consistent compressions.^8^ Other approaches to increase blood flow during resuscitation such as abdominal binding have been investigated and may be beneficial,^9^ although they have not been integrated into clinical practice. Beyond mechanical approaches, standard protocols include administration of epinephrine and anti-arrhythmic drugs to increase peripheral vascular resistance, eliminate arrhythmias, and raise mean blood pressure.^10^ However, in spite of advances in techniques and technology, CPR has an overall success rate between 10% and 20%,^1^,^11^–^13^ and is often complicated by pulmonary edema, rib fracture, gastric dilation, and sternal fracture.^14^

### ECMO—Fundamental Principles

Extracorporeal membrane oxygenation (ECMO) is a form of mechanical circulatory support that combines an extracorporeal blood pump with an oxygenator. The ECMO technique has traditionally been deployed for a variety of cardiopulmonary conditions and, more recently, sudden cardiac arrest.^15^

**Figure 1:**
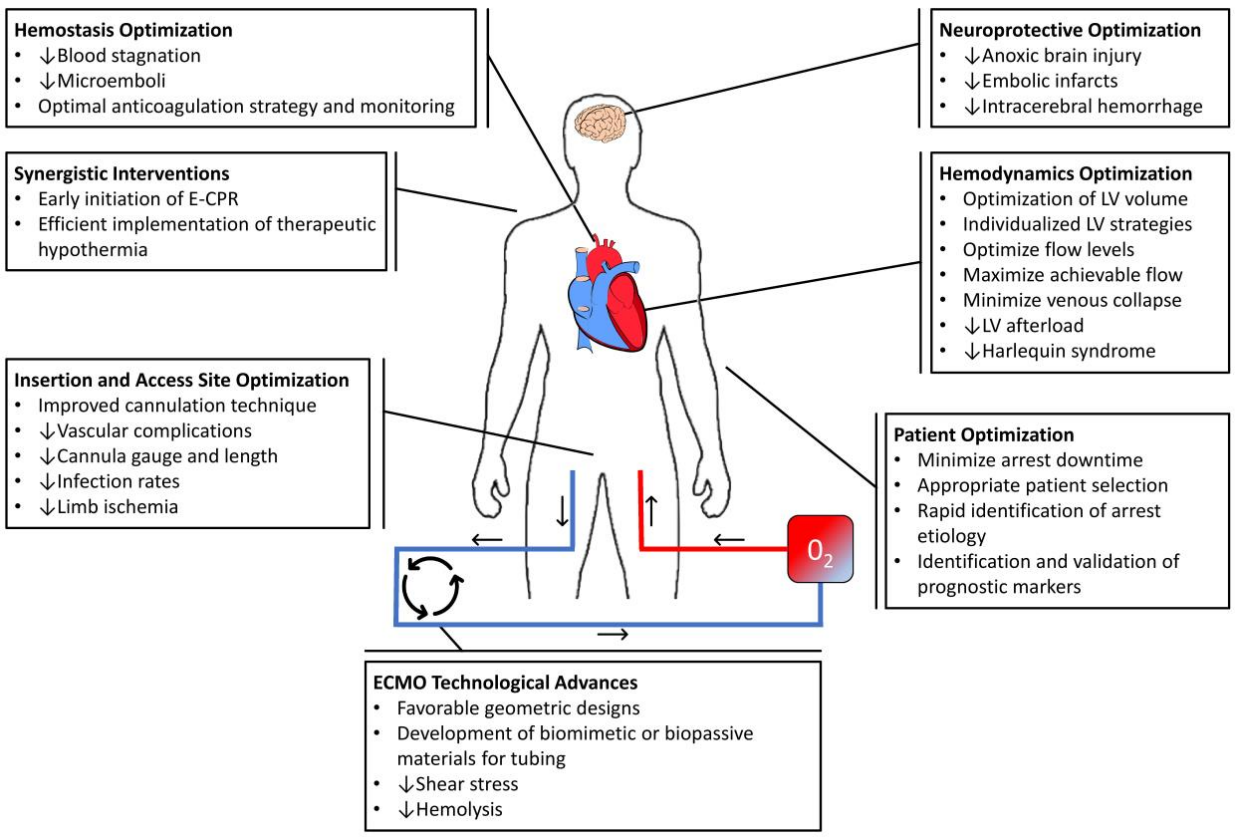
Schematic of VA-ECMO with Peripherally Inserted Cannula in the Right Femoral Vein and Left Femoral Artery The figure highlights the many areas of potential improvement and enhancement for ECMO and E-CPR to improve resuscitation efforts of sudden cardiac death.

There are two common configurations of ECMO which enable customization of support for each patient. In general, for respiratory support during which oxygenation is affected, such as with severe refractory acute respiratory distress syndrome, veno-venous ECMO (VV-ECMO) can be used to remove deoxygenated blood from the venous system, pass it through an oxygenator, then return the oxygenated blood into the circulation via the venous system. However, in cases of cardiac failure during which oxygenated blood is not adequately circulated throughout the body, additional support can be obtained through veno-arterial ECMO (VA-ECMO) (Figure 1). With VA-ECMO, venous blood is removed from the venous system and then pumped through an oxygenator with sufficient pressure to be returned to the body’s arterial tree via a peripherally (usually the femoral artery) or centrally placed cannula with adequate flow to restore end-organ perfusion.

At present, initiation and management of ECMO can be cumbersome, as it requires a skilled and experienced provider to cannulate, as well as systemic anticoagulation therapy, and a perfusionist dedicated to bedside management. When applied for refractory cardiac failure, ECMO results in approximately 60% survival in pediatric patients and around 40% in adults.^16^ Adverse events associated with ECMO include surgical and cannula site hemorrhages, infection, renal failure, hyperbilirubinemia, and oxygenator mechanical failure.^16^ Other common complications include clot and fibrin formation, hemolysis, air embolism, left ventricle overloading, Harlequin syndrome, and limb ischemia.^17^

### ECMO for Sudden Cardiac Arrest and Extracorporeal CPR

Recent studies have identified ECMO as a reasonable alternative to traditional CPR in the setting of cardiac arrest, when appropriate resources are available. The use of VA-ECMO in patients who suddenly experience a loss of pulse due to compromised cardiac mechanical activity is known as extracorporeal CPR (E-CPR).^18^ Previously published studies typically evaluate the effectiveness of E-CPR based on survival to discharge and long-term neurologic function, where out-of-hospital cardiac arrest (OHCA) and in-hospital cardiac arrest (IHCA) are considered independently.

Extracorporeal membrane oxygenation (ECMO) can be used in sudden cardiac arrest to restore blood flow when the heart has little or no intrinsic mechanical activity. When applied for sudden cardiac arrest, ECMO can double survival rates relative to CPR, even after 50 minutes of arrest.^19^–^21^ Further, a recent study demonstrated a 100% survival in patients who had ECMO started within 30 minutes of arrest and 25% survival with ECMO started within 75 minutes.^22^ For perspective, in 88 patients experiencing IHCA, the average duration of conventional CPR (C-CPR) was 13 minutes when return of spontaneous circulation was achieved (*n*=48) versus 28 minutes when resuscitation was unsuccessful (*n*= 40).^23^

### E-CPR Compared to C-CPR

Evidence generally supports the use of E-CPR when compared to C-CPR. However, the evidence varies. One systematic review of 25 observational studies concluded that due to low quality of evidence across studies there was no clear advantage to E-CPR over manual or mechanical CPR,^24^ while another showed similar survivals for E-CPR and C-CPR when applied for OHCA.^25^ Conversely, a meta-analysis showed improved survival and favorable neurologic outcomes for E-CPR compared to C-CPR, particularly at 3 to 6 months after arrest.^26^ Another meta-analysis of 2,260 patients also favored E-CPR over C-CPR based on survival to discharge and long-term neurologic outcome.^27^ In another single study of 531 patients, 38 received E-CPR for non-shockable OHCA, and it was found that the 1–3-month survival and cerebral function outcomes were higher in the E-CPR group than in the C-CPR group.^28^ From a prospective, observational study of 454 OHCA patients, those treated with E-CPR had significantly higher rates of favorable neurologic scores at both 1 and 6 months post-arrest as compared to those treated with C-CPR.^29^ Via *post hoc* analysis of data from a prospective observational cohort, 48 OHCA patients were propensity-matched, with intact survival being significantly higher in the E-CPR group than in the C-CPR group.^30^ Patients were also propensity-matched in a retrospective observational study of 406 IHCA patients, and again a survival benefit was found with E-CPR relative to C-CPR.^31^

### E-CPR for OHCA versus IHCA

Studies comparing the effectiveness of E-CPR for OHCA versus IHCA are inconclusive. A systematic review of 15 OHCA studies and 7 IHCA studies concluded that evidence did not support or refute the use of E-CPR for either group.^24^ Another study in 77 patients found that outcomes were more favorable for IHCA than for OHCA patients, but the difference was explained by patient factors and the time delay in starting E-CPR.^32^ In 423 patients receiving E-CPR, favorable neurologic outcome rate was significantly higher in the IHCA group (34%) compared to the OHCA group (9%), although the latter had a significantly longer time from collapse to E-CPR.^33^ Survival differences were even more dramatic between IHCA (42%) and OHCA (15%) groups among 85 non-postcardiotomy patients; however, the finding is complicated by a significantly shorter C-CPR duration in the IHCA group.^34^

A systematic review of refractory OHCA found a survival rate of 22%, and 13% had satisfactory neurologic recovery,^35^ which is comparable to the 12% favorable neurologic rate in 260 E-CPR OHCAs.^29^ A prospective registry study of 525 E-CPR OHCAs reported 8% survival.^25^ A retrospective multi-center study involving 258 E-CPR OHCAs found that intensive care unit (ICU) survival was 24%, and 19% had favorable neurologic outcomes.^33^ Further, a retrospective chart review involving 20 E-CPR OHCAs due to ventricular fibrillation revealed a 95% sustained return of spontaneous circulation (ROSC), 50% survival at discharge, 50% survival 1 year after discharge, and 40% adequate neurologic function at discharge.^36^ A cohort study featuring 1,796 E-CPR OHCA patients reported 29% survival to discharge, and survival did not significantly change across cohorts^37^; this is similar to the rate described in a propensity-matched study of 24 E-CPR OHCA patients.^30^

### Predictors of E-CPR Success

Patient selection, time course of resuscitation, and patient age are likely determinants of E-CPR outcomes. A potential neurologic outcome improvement of 20% was demonstrated when patients were chosen based on being ≤65 years old, were witnessed having arrest and received bystander CPR, had no major comorbidities, and ECMO was initiated within 1 hour of arrest.^33^ A similar study of 25 patients reported 44% having favorable neurologic recovery, with patient selection based on the arrest being of cardiac or pulmonary cause, the arrest being witnessed, chest compressions begun within 10 minutes, the initial rhythm was ventricular fibrillation or ventricular tachycardia, a mechanical CPR device was available with the paramedics, and time from collapse to arrival at the hospital was less than 60 minutes.^38^ These findings of strict patient selection are reinforced by a meta-analysis that observed a negative trend in survival when manual CPR was done for more than 30 minutes,^39^ and another in which favorable neurologic survival decreased from more than 30% to around 15% when arrest to E-CPR time exceeded 40 minutes.^40^ Patient age was also associated with decreased 1-month survival in another study that concluded that patients who are older than 70 may not be suitable candidates for E-CPR.^41^

The identification and validation of prognostic markers of E-CPR outcomes would be valuable to inform patient selection. However, one review specifies that prognostic markers are not available for OHCA E-CPR,^42^ while another review described shockable rhythms, witnessed events, and reversible cause of arrest as favorable prognostic factors.^35^ Other independent prognostic factors determining favorable cerebral function outcomes may also include E-CPR use and time from arrest to hospital arrival.^28^ A study in 10 OHCA patients suggests pupil diameter ≥6 centimeters as a possible contra-indication of E-CPR.^30^

## LIMITATIONS OF ECMO APPLICATION

### Resource Requirements

While mechanical circulatory support with ECMO continues to be a promising therapy to improve resuscitation efforts, current systems are resource-intensive and have substantial morbidity which limits more widespread use. A perfusionist is required in ECMO to prime, de-bubble, and manage the system, and this is generally not available in an emergency. Surgeons, who may also not be immediately available, are needed to place the large-bore cannula that enables venous withdrawal and allows adequate flow without venous collapse in adults. In addition, the large cannula size increases vascular complications, especially limb ischemia and bleeding around the insertion site.^43^ Cannula size is even more significant in pediatrics, where adequate flows may be limited by the need to use small cannulas due to the relatively smaller blood vessels.

### Expertise and Training

At this time, guidelines for training and continuation of ECMO specialists suggest that physicians “successfully complete institutional training requirements for clinical specialists.”^44^ As such, there are no universal requirements for physicians who intend to use ECMO on their patients. While this is beneficial in that it is less restrictive and can lead to further patient care with ECMO, it can be potentially harmful if physicians without significant surgical/interventional/ECMO experience run into device- and management-related complications.

### ECMO Availability

From 2006 to 2011 there was a 433% increase in ECMO use in adults in the United States.^45^ Similar trends have been shown globally with a 5-fold increase in the number of Extracorporeal Life Support Organization (ELSO) reporting centers between 1986 and 2015, with higher implementation rates and expanded indication criteria.^16^ However, ECMO remains predominantly available in large academic medical centers located in urban areas. Nonetheless, as ECMO becomes more available, a decrease in the traditional limiting factors of ECMO utilization such as personnel time and cost is anticipated. Improvements are expected to continue as both the number of available centers and the indications for ECMO grow.

## KNOWLEDGE GAPS IN THE USE OF ECMO

There are many gaps in knowledge that, if filled, could dramatically enhance the future success of ECMO, particularly when applied for cardiac arrest.

### Determinants of Adequate Perfusion

The goal of VA-ECMO is to provide adequate perfusion to achieve hemodynamic stability and return and maintain oxygenation of vital organs. There are various hemodynamic parameters that must be considered when assessing ECMO flow. To reach adequate arterial oxygenation, ECMO flows generally need to reach >60% of normal cardiac output. This is achieved through the use of various types of blood pumps that provide pressure to drive blood through the circuit. As such, the revolutions per minute (RPM), flow (L/min), inflow and outflow pressures, and mixed venous oxygen saturation (SvO_2_) are all actively monitored. Changes in these parameters can be indicative of both intrinsic and extrinsic issues of the circuit. For example, in the setting of a stable RPM, a flow rate drop may be reflective of decreased preload (hypovolemia, bleeding, tension pneumothorax) or increased afterload (membrane thrombus, arterial cannula kink, or elevated systemic vascular resistance).^46^

Mean arterial pressure (MAP) is another surrogate marker of perfusion that is carefully monitored in all patients with hemodynamic compromise. The MAP can be roughly approximated as a function of diastolic and systolic pressures, or alternatively as a surrogate of cardiac output, central venous pressure, and systemic vascular resistance. In VA-ECMO, MAP is determined by a combination of native cardiac function and circuit pump output, which can occur with or without pulsatility depending on the relative driving force. Therefore, flow is adjusted based on multiple parameters including MAP, intrinsic cardiac function, systemic vascular resistance, or need for additional support with inotropic agents or other mechanical circulatory support devices.

Veno-arterial ECMO flows begin at 30 mL/kg/minute of ideal body weight with a desired central venous oxygen saturation >70%. However, in general, target perfusion for adults is 60 mL/kg/min.^44^ Gas flow, also known as sweep, controls how much carbon dioxide is removed, and is adjusted to maintain blood pH and partial pressure of carbon dioxide at 7.40 and 40 mmHg, respectively. Of the available parameters used to assess optimal systemic perfusion, the most objective and clinically relevant are the oxygen saturation (SpO_2_) and lactate level as they directly correlate with tissue perfusion. Ideal settings will balance oxygen delivery and absorption, as reflected by a venous oxygen saturation >70% and serum lactate level less than 2.2 mmol/L or those trending towards normal. If SvO_2_ and lactate do not recover within a reasonable time period while on ECMO, it is possible that either ECMO may not be indicated (i.e. high output septic shock) or there is poor delivery of oxygen, which may require increased flows or transfusion.^47^

While hemodynamic and physiologic indicators of perfusion are essential for ECMO management strategies, there are substantial limitations. First, these indicators are based on population averages and therefore may not be sufficient for an individual patient. Second, these indicators are specific to whole-body physiology and thus do not represent what a specific organ requires versus what it is receiving. Finally, oxygen and blood flow delivery demands may shift based on many factors such as patient alertness and blood shunting to the gut if the patient is able to eat while on ECMO. While the latter is not relevant to ECMO applied for sudden cardiac arrest, all these limitations stem from not knowing tissue-level, organ-specific, real-time perfusion demand versus supply.

### ECMO, Left Ventricular Volume, and Left Ventricular Venting

Extracorporeal membrane oxygenation (ECMO) has profound implications for central arterial and left ventricular (LV) dynamics. As VA-ECMO displaces blood from a large venous reservoir to the arterial circulation, the patient’s volume status and intrinsic ventricular function are important components that impact LV volume. For example, if the patient has reduced preload (right atrial pressure is reduced), then, with ECMO initiation, the left ventricle will have a reduction in preload and relatively diminished LV volume. However, because VA-ECMO is often utilized in cardiogenic shock where there may be concomitant congestion, additional pressurization of the arterial system will increase systemic blood pressure. If the native LV function is preserved, LV systolic pressures will increase and overcome the LV afterload leading to an ejection of blood through the aortic valve. Additionally, if LV contractility is adequate, increases in flow and LV systolic pressure will not come at the expense of the LV diastolic pressure. However, if the native LV function is compromised, both LV systolic and diastolic pressures will increase with a concurrent reduction in stroke volume as the left ventricle fails to pump effectively against increased afterload imposed by an extracorporeal pump and pressurized arterial tree. This increase in LV afterload increases LV and left atrial wall stress, myocardial oxygen consumption, and may worsen pulmonary congestion, acute lung injury, and pulmonary hemorrhage, thereby worsening cardiopulmonary function and initiating a vicious cycle of mechanically driven injury.^48^,^49^ Paradoxically, high afterload may result in diminished coronary flow due to LV distension and subsequently increased coronary resistance.^50^

In an attempt to mitigate increased LV diastolic pressures and LV volume, there have been several proposed methods for what has been termed *LV venting* to assist the ailing left ventricle in the setting of ECMO. Such strategies involve inotropic support, passive venting with atrial septostomy, central or peripheral surgical venting, trans-septal inflow catheters or cannulas, intra-aortic balloon pump, and Impella trans-aortic axial flow pumps (Abiomed Inc., Danvers, MA, USA). A recent systematic review found that LV venting, especially if done early (<12 hours after initiation of ECMO), appears to be associated with increased success of weaning and reduced short-term mortality.^51^

Left ventricular volume also depends on the level of ECMO flow. In a previously validated model of the cardiovascular system that generated pressure–volume loops and Starling curves, the addition of VA-ECMO did not affect LV contractile function but, in the setting of a failing heart, increased afterload.^52^ This increase in afterload has similar hemodynamic consequences seen with increases in systemic vascular resistance.^52^ Also, in cases with fixed systemic vascular and LV contractility, LV distension is based on the Starling principle.^53^ Therefore, in patients with poor LV contractile function, with increases in ECMO flow, one would expect increased afterload, decreased native stroke volume, and increased LV volume, leading to increased pulmonary wedge pressures, and pulmonary congestion. It has been shown that as ECMO is initiated and then increased stepwise from 1.4 L/min to 3.0 L/min to 4.5 L/min, the primary hemodynamic effect is increased LV afterload.^53^ Another study showed that the extent of LV distension was inversely related to recovery.^54^ Thus, there exists an optimum for ECMO when applied for sudden cardiac death whereby flow needs to be high enough to provide sufficient perfusion to ischemically vulnerable organs while not providing so much flow that afterload is overwhelmingly high, preventing myocardial recovery.

While it is appreciated that ECMO flows often lead to overloading of the left ventricle to the point of limiting or even preventing ejection, there are many unknown aspects of this relationship. For example, it is not known which strategy is best for venting or what volume of LV distension requires venting. Also, the influence that time and the volume–time profile have on the detrimental manifestations of low native ejection is not known.

### Afterload, Stagnation, and Microemboli

Understanding arterial and ventricular dynamics during ECMO is important because overloading of the ventricle leads to depressed ejection, which may in turn result in blood stagnation and microemboli^55^ that may ultimately cause end-organ damage. However, the only technique able to detect microembolic signals in intracranial arteries in real-time is multi-gated transcranial Doppler. A single-center observational prospective study in patients who underwent ECMO and had transcranial Doppler revealed that microemboli were present in both VA- and VV-ECMO configurations, but these emboli did not seem to influence clinical outcome.^56^ The VA-ECMO population had a significantly higher number of emboli, and more emboli were noted in patients with extremely low native cardiac output, which suggests that the microemboli came from the ECMO circuit because cerebral flow was largely from the circuit rather than native cardiac output. However, there is also the possibility that, in cases of depressed cardiac function, microemboli can originate in the left heart.^56^ Nonetheless, this particular study did not find a significant correlation between neurological defects and the presence of microembolic signals.^36^ Veno-arterial ECMO may also have a higher risk of neurological complications because the oxygenator outlet is a potential source of emboli and returns blood directly into the central arteries. Additional sources of microemboli include the arterial line during the cannulation and decannulation procedures and thrombosis development within the circuit, all of which may lead to cerebral infarcts.^55^,^56^ These sources result in current cerebrovascular complication rates in ECMO patients of about 7%.^56^

### Anticoagulation

Bleeding and clotting are the two most common VA-ECMO complications, with significant clot formation within the circuit or oxygenator occurring in approximately 10% of adult cases.^16^ Thrombotic events including stroke (3.8%–6.8%) and limb ischemia (3.6%) are less frequent, while hemorrhagic complications occur in 27%–44% of patients and include a 2.2% risk of intracranial hemorrhage.^17^,^57^ Nonetheless, there is currently not an optimal strategy for anticoagulation management for patients on VA-ECMO.^57^ One study compared two anticoagulation targets (activated coagulation time [ACT] target 140–160 seconds versus 180–220 seconds) and found a significantly higher amount of cannula site bleeding, bleeding-induced death, and major bleeding events in the higher-target group.^58^ The optimal test for assessing anticoagulation status in patients remains unclear as well. Studies have assessed ACT and activated partial thromboplastin time (aPTT), which weakly correlate with anti-Xa levels and heparin activity. Anti-Xa levels in VA-ECMO also remain unknown, and a measure of this activity is not available at many centers.^57^ Current guidelines suggest an unfractionated heparin bolus of 50–100 IU/kg at the time of cannulation, and titration of unfractionated heparin to an activated clotting time or activated partial thromboplastin time at least 1.5 times the upper limit of normal, or anti-factor Xa activity levels of 0.5 IU/mL.^59^ Direct thrombin inhibitor (argatroban or bivalirudin) may be used in the event of heparin-induced thrombocytopenia, a rare condition characterized by multiple white arterial thrombi and platelet count dropping below 10,000 without another etiology for thrombocytopenia.

## OPPORTUNITIES FOR TECHNOLOGIC IMPROVEMENT

To make a significant impact on cardiac arrest resuscitation outcomes, there is the obvious need for collaborative efforts between clinicians, engineers, and scientists to bridge physiologic and medical gaps. Improvements to ECMO technology has tremendous potential to bridge these gaps and further optimize ECMO as an E-CPR strategy for cardiac arrest resuscitation. Potential concepts for technology improvements are included here.

### Thrombosis and Blood Damage

As with other blood-wetted circulatory support technologies such as ventricular assist devices, ECMO systems are innately prone to complications such as thrombosis, hemolysis, and percutaneous site infection. Therefore, advances made in blood-wetted biomaterials and hemodynamically favorable geometric designs can benefit prospective ECMO circuits.

A general strategy to limit thrombosis and enhance overall hemocompatibility in blood-wetted devices involves minimizing artificial surface area. Less surface area means fewer possible niduses for clot formation. In the context of ECMO, the largest artificial surface area is associated with the oxygenator, which is likely not available for minimization because oxygen diffusion also depends on surface area. Previous attempts to miniaturize the ECMO oxygenator experienced difficulties in one preclinical study.^60^ However, in another preclinical study, miniaturized ECMO systems demonstrated similar hemocompatibility relative to a standard ECMO configuration,^61^ and in coronary artery bypass grafting patients a miniaturized ECMO led to better end-organ outcomes than standard ECMO.^62^

Beyond reducing surface area, the blood-wetted surfaces have a role in thrombosis. Current ECMO circuits commonly utilize standard PVC tubing^63^; however, surface modifications are being investigated to improve hemocompatibility via biomimetic, biopassive, or endothelialized blood–surface interfaces.^64^ Such modifications have shown promise in preclinical and clinical studies^65^,^66^ and may reduce systemic anticoagulation^67^ but are yet to be incorporated as common ECMO practice.

Another approach to limit thrombosis and hemolysis entails pump design via computational fluid dynamics (CFD), which is an advanced computational technique that enables the three-dimensional prediction of flow field characteristics (including streamlines, residence time, shear stress, and shear rate) around both stationary and moving solid structures. Using CFD enables continuous flow axial and centrifugal blood pumps to be designed and optimized so that zones of stasis and profiles of shear stress and shear rate that activate platelets and cause hemolysis are avoided.^68^ Axial and centrifugal devices contrast with the roller pumps that are still common to ECMO circuits and in which tube compression may even more dramatically damage blood. Although proven to be highly successful in ventricular assist device design, in limited ECMO studies continuous flow pumps have not demonstrated a convincing advantage over roller pumps.^69^–^71^ However, these comparisons do not include the most advanced continuous flow pumps, and thus the corresponding methods have been questioned.^72^,^73^

### Circuit Volume

It is also important to minimize the volume required to prime the ECMO circuit. Saline priming dilutes red blood cell concentration, and thus higher flows are necessary to provide the same perfusion than with less priming volume. Higher flows cause more blood damage via relatively increased shear rates and shear stress, therefore promoting thrombosis and hemolysis. Studies on flow velocity have also shown that high ECMO flow can lead to high wall shear stress and hypertension, ultimately leading to vascular complications and acute limb ischemia.^74^

### Real-time Cerebral Blood Flow via MRI

Another technology that may prove highly valuable to ECMO optimization is magnetic resonance imaging (MRI) because it is capable of evaluating cerebral blood flow and cerebral rate of oxygen consumption in real time. Specifically, brain blood flow can be measured directly,^75^,^76^ whereas oxygen extraction fraction can be estimated via arteriovenous oxygen content, and then cerebral metabolic rate of oxygen can be estimated from blood flow and oxygen extraction fraction.^77^ Therefore, flow rates can be titrated to cerebral oxygen homeostasis non-invasively.^78^–^80^ Nonetheless, current ECMO system configurations are not MRI-compatible, although a preclinical study indicates that cerebral structure can be evaluated via an MRI-adapted ECMO system.^81^

## OPPORTUNITIES FOR SYNERGY

When applied for resuscitation purposes, ECMO is often initiated after C-CPR, and in this manner the two therapies are complementary. Additional opportunities exist to optimize patient outcomes via synergistic therapies. Two additional examples of synergy involving ECMO are included here.

### E-CPR and Therapeutic Hypothermia

The ECMO outcomes for sudden cardiac arrest may be improved with simultaneous therapeutic hypothermia. Beyond ECMO, therapeutic hypothermia is one of the few treatments that enhances cardiac and neurological function and survival outcomes. However, it takes hours to reach the targeted temperature even though a drop of only a few degrees is sought (commonly from 37°C to 34–36°C). If active cooling is delayed by just 20 minutes, survival benefit is lost, suggesting that an important therapeutic window exists during CPR for affecting sudden cardiac arrest outcome.^82^ Similarly, shorter arrest-to-ECMO times are associated with improved survival in refractory cardiac arrest.^83^,^84^ Thus, there is a potential benefit to devising protocols that employ E-CPR and therapeutic strategies in a timely manner.

The CHEER trial is a single-center, prospective, observational study for selected patients with refractory IHCA and OHCA. Patients in this study received mechanical CPR, E-CPR, and rapid intravenous administration of 30 mL/kg of ice-cold saline to induce intra-arrest therapeutic hypothermia. In that study, survival to hospital discharge was 60% in patients with refractory IHCA and 45% for OHCA.^84^

In a porcine model of ventricular fibrillation arrest with ECMO, 2-hour therapeutic hypothermia during E-CPR offers an equal resuscitation success rate, but did not preserve post-arrest cardiac function nor reduce the magnitude of myocardial injury, compared to normothermic E-CPR.^85^ Some studies evaluated the combined use of E-CPR and therapeutic hypothermia in adult cardiac arrest patients and/or compared therapeutic hypothermia treatment with no therapeutic hypothermia induction. However, the sample size of those studies was limited, the proportion of patients receiving therapeutic hypothermia ranges varied, and no conclusive result was derived as to whether there was a benefit of therapeutic hypothermia treatment in cardiac patients undergoing E-CPR.^86^ A systematic review with meta-analysis comparing neurological outcomes and survival between therapeutic hypothermia treatment at 32–34°C and any other temperature controls, including no therapeutic hypothermia induction and alternative targeted temperatures range (>34°C, ≤36°C), in adult cardiac arrest patients receiving E-CPR showed an association with favorable neurologic outcomes and survival.^86^ Though not dedicated to assessing the relative contribution of therapeutic hypothermia, several other studies report favorable neurologic outcomes when E-CPR is utilized with active cooling of OHCA patients.^29^,^30^

### ECMO for Sudden Cardiac Arrest and Transplant Donors

In addition to improvement of cardiac arrest resuscitation outcomes, there is potential to address the organ shortage in the transplantation community.^87^ Data from the USA show that there were 120,000 patients waiting for transplants as of April 2020, while in 2019 only 39,719 transplants were performed from 19,253 donors, and 15 patients died each day waiting for a transplant.^88^ More than half of cardiac arrest victims do not have ROSC, even with use of ECMO.^19^–^21^,^89^ In addition, a substantial number of patients with ROSC will be brain dead, despite the use of ECMO. Patients with ongoing ECMO, but without ROSC, or with brain death, represent a large pool of viable donors, since it has been shown that kidneys and livers from donors on ECMO, but with brain death, had similar survival to those not on ECMO.^90^ In a systematic review including 833 patients receiving E-CPR, 650 were non-survivors, and, of those, 88 were potential organ donors, while 17 served as organ donors.^35^ In another study of 423 E-CPR patients, 321 were non-survivors, and 23 were organ donors.^33^

## CONCLUSION

There are abundant opportunities to improve future sudden cardiac arrest outcomes via progress in research and technology of ECMO, and through the synergy of CPR, ECMO, and therapeutic hypothermia. These advances will ultimately result in ECMO being more commonly utilized for sudden cardiac arrest at a larger distribution of clinical sites by a more broadly trained pool of providers. Organ transplant recipients may also benefit from expanded ECMO application that includes sudden cardiac arrest.

## Abbreviations

ACT: activated coagulation time
aPTT: activated partial thromboplastin time
C-CPR: conventional CPR
CFD: computational fluid dynamics
CPR: cardiopulmonary resuscitation
ECMO: extracorporeal membrane oxygenation
E-CPR: extracorporeal CPR
ELSO: Extracorporeal Life Support Organization
IHCA: in-hospital cardiac arrest
LV: left ventricular
MAP: mean arterial pressure
OHCA: out-of-hospital cardiac arrest
ROSC: return of spontaneous circulation
VA-ECMO: veno-arterial ECMO
VV-ECMO: veno-venous ECMO

## References

[1] Benjamin EJ, Muntner P, Alonso A (2019). Heart disease and stroke statistics-2019 update: a report from the American Heart Association. Circulation.

[2] Adabag AS, Luepker RV, Roger VL, Gersh BJ (2010). Sudden cardiac death: epidemiology and risk factors. Nat Rev Cardiol.

[3] Halperin HR, Guerci AD, Chandra N (1986). Vest inflation without simultaneous ventilation during cardiac arrest in dogs: improved survival from prolonged cardiopulmonary resuscitation. Circulation.

[4] Halperin HR, Tsitlik JE, Gelfand M (1993). A preliminary study of cardiopulmonary resuscitation by circumferential compression of the chest with use of a pneumatic vest. N Engl J Med.

[5] Halperin HR, Tsitlik JE, Guerci AD (1986). Determinants of blood flow to vital organs during cardiopulmonary resuscitation in dogs. Circulation.

[6] Hightower D, Thomas SH, Stone CK, Dunn K, March JA (1995). Decay in quality of closed-chest compressions over time. Ann Emerg Med.

[7] Ashton A, McCluskey A, Gwinnutt CL, Keenan AM (2002). Effect of rescuer fatigue on performance of continuous external chest compressions over 3 min. Resuscitation.

[8] Fox J, Fiechter R, Gerstl P (2013). Mechanical versus manual chest compression CPR under ground ambulance transport conditions. Acute Card Care.

[9] Chandra N, Snyder LD, Weisfeldt ML (1981). Abdominal binding during cardiopulmonary resuscitation in man. JAMA.

[10] Zhong JQ, Dorian P (2005). Epinephrine and vasopressin during cardiopulmonary resuscitation. Resuscitation.

[11] Weaver WD (1991). Resuscitation outside the hospital -- What’s lacking?. N Engl J Med.

[12] Meaney PA, Nadkarni VM, Kern KB, Indik JH, Halperin HR, Berg RA (2010). Rhythms and outcomes of adult in-hospital cardiac arrest. Crit Care Med.

[13] Bedell SE, Delbanco TL, Cook EF, Epstein FH (1983). Survival after cardiopulmonary resuscitation in the hospital. N Engl J Med.

[14] Nagel EL, Fine EG, Krischer JP, Davis JH (1981). Complications of CPR Crit Care Med.

[15] Patel AR, Patel AR, Singh S, Singh S, Khawaja I (2019). Applied uses of extracorporeal membrane oxygenation therapy. Cureus.

[16] Thiagarajan RR, Barbaro RP, Rycus PT (2017). Extracorporeal Life Support Organization registry international report 2016. ASAIO J.

[17] Eckman PM, Katz JN, El Banayosy A, Bohula EA, Sun B, van Diepen S (2019). Veno-arterial extracorporeal membrane oxygenation for cardiogenic shock: an introduction for the busy clinician. Circulation.

[18] Pappalardo F, Montisci A (2017). What is extracorporeal cardiopulmonary resuscitation?. J Thorac Dis.

[19] Chen YS, Yu HY, Huang SC (2008). Extracorporeal membrane oxygenation support can extend the duration of cardiopulmonary resuscitation. Crit Care Med.

[20] Nagao K, Kikushima K, Watanabe K (2010). Early induction of hypothermia during cardiac arrest improves neurological outcomes in patients with out-of-hospital cardiac arrest who undergo emergency cardiopulmonary bypass and percutaneous coronary intervention. Circ J.

[21] Athanasuleas CL, Buckberg GD, Allen BS, Beyersdorf F, Kirsh MM (2006). Sudden cardiac death: directing the scope of resuscitation towards the heart and brain. Resuscitation.

[22] Bartos JA, Carlson K, Carlson C (2018). Surviving refractory out-of-hospital ventricular fibrillation cardiac arrest: critical care and extracorporeal membrane oxygenation management. Resuscitation.

[23] Cheema MA, Ullah W, Abdullah HMA, Haq S, Ahmad A, Balaratna A (2019). Duration of in-hospital cardiopulmonary resuscitation and its effect on survival. Indian Heart J.

[24] Holmberg MJ, Geri G, Wiberg S (2018). Extracorporeal cardiopulmonary resuscitation for cardiac arrest: a systematic review. Resuscitation.

[25] Bougouin W, Dumas F, Lamhaut L (2019). Extracorporeal cardiopulmonary resuscitation in out-of-hospital cardiac arrest: a registry study. Eur Heart J.

[26] Kim SJ, Kim HJ, Lee HY, Ahn HS, Lee SW (2016). Comparing extracorporeal cardiopulmonary resuscitation with conventional cardiopulmonary resuscitation: a meta-analysis. Resuscitation.

[27] Wang GN, Chen XF, Qiao L (2017). Comparison of extracorporeal and conventional cardiopulmonary resuscitation: a meta-analysis of 2 260 patients with cardiac arrest. World J Emerg Med.

[28] Yoshida T, Fujitani S, Wakatake H (2020). Exploratory observational study of extracorporeal cardiopulmonary resuscitation for nonshockable out-of-hospital cardiac arrest occurring after an emergency medical services arrival: SOS-KANTO 2012 Study Report. J Emerg Med.

[29] Sakamoto T, Morimura N, Nagao K (2014). Extracorporeal cardiopulmonary resuscitation versus conventional cardiopulmonary resuscitation in adults with out-of-hospital cardiac arrest: a prospective observational study. Resuscitation.

[30] Maekawa K, Tanno K, Hase M, Mori K, Asai Y (2013). Extracorporeal cardiopulmonary resuscitation for patients with out-of-hospital cardiac arrest of cardiac origin: a propensity-matched study and predictor analysis. Crit Care Med.

[31] Shin TG, Choi JH, Jo IJ (2011). Extracorporeal cardiopulmonary resuscitation in patients with inhospital cardiac arrest: a comparison with conventional cardiopulmonary resuscitation. Crit Care Med.

[32] Kagawa E, Inoue I, Kawagoe T (2010). Assessment of outcomes and differences between in- and out-of-hospital cardiac arrest patients treated with cardiopulmonary resuscitation using extracorporeal life support. Resuscitation.

[33] Lunz D, Calabro L, Belliato M (2020). Extracorporeal membrane oxygenation for refractory cardiac arrest: a retrospective multicenter study. Intensive Care Med.

[34] Haneya A, Philipp A, Diez C (2012). A 5-year experience with cardiopulmonary resuscitation using extracorporeal life support in non-postcardiotomy patients with cardiac arrest. Resuscitation.

[35] Ortega-Deballon I, Hornby L, Shemie SD, Bhanji F, Guadagno E (2016). Extracorporeal resuscitation for refractory out-of-hospital cardiac arrest in adults: a systematic review of international practices and outcomes. Resuscitation.

[36] Siao FY, Chiu CC, Chiu CW (2015). Managing cardiac arrest with refractory ventricular fibrillation in the emergency department: conventional cardiopulmonary resuscitation versus extracorporeal cardiopulmonary resuscitation. Resuscitation.

[37] Richardson AS, Schmidt M, Bailey M, Pellegrino VA, Rycus PT, Pilcher DV (2017). ECMO cardio-pulmonary resuscitation (ECPR), trends in survival from an international multicentre cohort study over 12-years. Resuscitation.

[38] Dennis M, Buscher H, Gattas D (2020). Prospective observational study of mechanical cardiopulmonary resuscitation, extracorporeal membrane oxygenation and early reperfusion for refractory cardiac arrest in Sydney: the 2CHEER study. Crit Care Resusc.

[39] Cardarelli MG, Young AJ, Griffith B (2009). Use of extractorporeal membrane oxygenation for adults in cardiac arrest (E-CPR): a meta-analysis of observational studies. ASAIO J.

[40] Yukawa T, Kashiura M, Sugiyama K, Tanabe T, Hamabe Y (2017). Neurological outcomes and duration from cardiac arrest to the initiation of extracorporeal membrane oxygenation in patients with out-of-hospital cardiac arrest: a retrospective study. Scand J Trauma Resusc Emerg Med.

[41] Goto T, Morita S, Kitamura T (2018). Impact of extracorporeal cardiopulmonary resuscitation on outcomes of elderly patients who had out-of-hospital cardiac arrests: a single-centre retrospective analysis. BMJ Open.

[42] Inoue A, Hifumi T, Sakamoto T, Kuroda Y (2020). Extracorporeal cardiopulmonary resuscitation for out-of-hospital cardiac arrest in adult patients. J Am Heart Assoc.

[43] Kim J, Cho YH, Sung K (2019). Impact of cannula size on clinical outcomes in peripheral venoarterial extracorporeal membrane oxygenation. ASAIO J.

[44] Extracorporeal Life Support Organization (ELSO) (2013). Guidelines for Adult Cardiac Failure Supplement. Version 1.3.

[45] Sauer CM, Yuh DD, Bonde P (2015). Extracorporeal membrane oxygenation use has increased by 433% in adults in the United States from 2006 to 2011. ASAIO J.

[46] Chung M, Shiloh AL, Carlese A (2014). Monitoring of the adult patient on venoarterial extracorporeal membrane oxygenation. ScientificWorldJournal.

[47] Choi MS, Sung K, Cho YH (2019). Clinical pearls of venoarterial extracorporeal membrane oxygenation for cardiogenic shock. Korean Circ J.

[48] Rao P, Khalpey Z, Smith R, Burkhoff D, Kociol RD (2018). Venoarterial extracorporeal membrane oxygenation for cardiogenic shock and cardiac arrest. Circ Heart Fail.

[49] Kapur NK, Davila CD, Chweich H (2019). Protecting the vulnerable left ventricle: the art of unloading with VA-ECMO. Circ Heart Fail.

[50] Rajagopal K (2019). Left ventricular distension in veno-arterial extracorporeal membrane oxygenation: from mechanics to therapies. ASAIO J.

[51] Al-Fares AA, Randhawa VK, Englesakis M (2019). Optimal strategy and timing of left ventricular venting during veno-arterial extracorporeal life support for adults in cardiogenic shock: a systematic review and meta-analysis. Circ Heart Fail.

[52] Dickstein ML (2018). The starling relationship and veno-arterial ECMO: ventricular distension explained. ASAIO J.

[53] Burkhoff D, Sayer G, Doshi D, Uriel N (2015). Hemodynamics of mechanical circulatory support. J Am Coll Cardiol.

[54] Truby LK, Takeda K, Mauro C (2017). Incidence and implications of left ventricular distention during venoarterial extracorporeal membrane oxygenation support. ASAIO J.

[55] Makdisi G, Hashmi ZA, Wozniak TC, Wang IW (2015). Left ventricular thrombus associated with arteriovenous extra corporeal membrane oxygenation. J Thorac Dis.

[56] Marinoni M, Migliaccio ML, Trapani S (2016). Cerebral microemboli detected by transcranial doppler in patients treated with extracorporeal membrane oxygenation. Acta Anaesthesiol Scand.

[57] Sy E, Sklar MC, Lequier L, Fan E, Kanji HD (2017). Anticoagulation practices and the prevalence of major bleeding, thromboembolic events, and mortality in venoarterial extracorporeal membrane oxygenation: a systematic review and meta-analysis. J Crit Care.

[58] Yeo HJ, Kim DH, Jeon D, Kim YS, Cho WH (2015). Low-dose heparin during extracorporeal membrane oxygenation treatment in adults. Intensive Care Med.

[59] Extracorporeal Life Support Organization (ELSO) (2014). ELSO Anticoagulation Guidelines.

[60] Kopp R, Bensberg R, Arens J (2011). A miniaturized extracorporeal membrane oxygenator with integrated rotary blood pump: preclinical in vivo testing. ASAIO J.

[61] Kopp R, Bensberg R, Henzler D (2010). Hemocompatibility of a miniaturized extracorporeal membrane oxygenation and a pumpless interventional lung assist in experimental lung injury. Artif Organs.

[62] Wiesenack C, Liebold A, Philipp A (2004). Four years’ experience with a miniaturized extracorporeal circulation system and its influence on clinical outcome. Artif Organs.

[63] Sarkar M, Prabhu V (2017). Basics of cardiopulmonary bypass. Indian J Anaesth.

[64] Ontaneda A, Annich GM (2018). Novel surfaces in extracorporeal membrane oxygenation circuits. Front Med (Lausanne).

[65] Thelin S, Bagge L, Hultman J, Borowiec J, Nilsson L, Thorelius J (1991). Heparin-coated cardiopulmonary bypass circuits reduce blood cell trauma. Eur J Cardiothorac Surg.

[66] Ranucci M, Mazzucco A, Pessotto R (1999). Heparin-coated circuits for high-risk patents: a multicenter, prospective, randomized trial. Ann Thorac Surg.

[67] Ovrum E, Tangen G, Oystese R, Ringdal MA, Istad R (2001). Comparison of two heparin-coated extracorporeal circuits with reduced systemic anticoagulation in routine coronary artery bypass operations. J Thorac Cardiovasc Surg.

[68] Nishida M, Maruyama O, Kosaka R (2009). Hemocompatibility evaluation with experimental and computational fluid dynamic analyses for a monopivot circulatory assist pump. Artif Organs.

[69] Barrett CS, Jaggers JJ, Cook EF (2013). Pediatric ECMO outcomes: comparison of centrifugal versus roller blood pumps using propensity score matching. ASAIO J.

[70] O’Halloran CP, Thiagarajan RR, Yarlagadda VV (2019). Outcomes of infants supported with extracorporeal membrane oxygenation using centrifugal versus roller pumps: an analysis from the Extracorporeal Life Support Organization registry. Pediatr Crit Care Med.

[71] Moon YS, Ohtsubo S, Gomez MR, Moon JK, Nose Y (1996). Comparison of centrifugal and roller pump hemolysis rates at low flow. Artif Organs.

[72] Byrnes JW, Fiser RT (2013). Comparing outcomes in ECMO between roller and centrifugal pumps in the face of evolving technology. Ann Thorac Surg.

[73] Dalton HJ, Hoskote A (2019). There and back again: roller pumps versus centrifugal technology in infants on extracorporeal membrane oxygenation. Pediatr Crit Care Med.

[74] Bisdas T, Beutel G, Warnecke G (2011). Vascular complications in patients undergoing femoral cannulation for extracorporeal membrane oxygenation support. Ann Thorac Surg.

[75] Calamante F, Thomas DL, Pell GS, Wiersma J, Turner R (1999). Measuring cerebral blood flow using magnetic resonance imaging techniques. J Cereb Blood Flow Metab.

[76] Griffiths PD, Hoggard N, Dannels WR, Wilkinson ID (2001). In vivo measurement of cerebral blood flow: a review of methods and applications. Vasc Med.

[77] Kety SS, Schmidt CF (1948). The nitrous oxide method for the quantitative determination of cerebral blood flow in man: theory, procedure and normal values. J Clin Invest.

[78] Liu P, Xu F, Lu H (2013). Test-retest reproducibility of a rapid method to measure brain oxygen metabolism. Magn Reson Med.

[79] Wise RG, Harris AD, Stone AJ, Murphy K (2013). Measurement of OEF and absolute CMRO2: MRI-based methods using interleaved and combined hypercapnia and hyperoxia. Neuroimage.

[80] Rodgers ZB, Detre JA, Wehrli FW (2016). MRI-based methods for quantification of the cerebral metabolic rate of oxygen. J Cereb Blood Flow Metab.

[81] Lidegran MK, Frenckner BP, Mosskin M, Nordell B, Palmer K, Linden VB (2006). MRI of the brain and thorax during extracorporeal membrane oxygenation: preliminary report from a pig model. ASAIO J.

[82] Nagao K (2012). Therapeutic hypothermia following resuscitation. Curr Opin Crit Care.

[83] Ryu JA, Cho YH, Sung K (2015). Predictors of neurological outcomes after successful extracorporeal cardiopulmonary resuscitation. BMC Anesthesiol.

[84] Stub D, Bernard S, Pellegrino V (2015). Refractory cardiac arrest treated with mechanical CPR, hypothermia, ECMO and early reperfusion (the CHEER trial). Resuscitation.

[85] Bergan HA, Halvorsen PS, Skulstad H, Fosse E, Bugge JF (2016). Does therapeutic hypothermia during extracorporeal cardiopulmonary resuscitation preserve cardiac function?. J Transl Med.

[86] Chen X, Zhen Z, Na J, Wang Q, Gao L, Yuan Y (2020). Associations of therapeutic hypothermia with clinical outcomes in patients receiving ECPR after cardiac arrest: systematic review with meta-analysis. Scand J Trauma Resusc Emerg Med.

[87] Saidi RF, Hejazii Kenari SK (2014). Challenges of organ shortage for transplantation: solutions and opportunities. Int J Organ Transplant Med.

[88] U.S. Department of Health & Human Services (2018). Organ Procurement and transplantation Network: National Data. National Data - OPTN.

[89] Sabashnikov A, Djordjevic I, Deppe AC (2018). Managing traps and pitfalls during initial steps of an ECMO retrieval program using a miniaturized portable system: what have we learned from the first two years?. Artif Organs.

[90] Bronchard R, Durand L, Legeai C, Cohen J, Guerrini P, Bastien O (2017). Brain-dead donors on extracorporeal membrane oxygenation. Crit Care Med.

